# Potential Alternative Receptors for SARS-CoV-2-Induced Kidney Damage: TLR-4, KIM-1/TIM-1, and CD147

**DOI:** 10.31083/j.fbl2901008

**Published:** 2024-01-12

**Authors:** Nada J. Habeichi, Ghadir Amin, Bachir Lakkis, Rayane Kataya, Mathias Mericskay, George W. Booz, Fouad A. Zouein

**Affiliations:** 1 Department of Pharmacology and Toxicology, American University of Beirut Faculty of Medicine, 1107-2020 Beirut, Lebanon; 2 The Cardiovascular, Renal, and Metabolic Diseases Research Center of Excellence, American University of Beirut Medical Center, Riad El-Solh, 1107-2020 Beirut, Lebanon; 3 Department of Signaling and Cardiovascular Pathophysiology, University Paris Saclay, INSERM UMR_1180, 91400 Orsay, France; 4 MatriceLab Innove Laboratory, Immeuble Les Gemeaux, 94000 Creteil, France; 5 Department of Pharmacology and Toxicology, School of Medicine, University of Mississippi Medical Center, Jackson, MS 39216, USA; 6 Division of Cardiology, Department of Internal Medicine, American University of Beirut Medical Center, 1107-2020 Beirut, Lebanon

**Keywords:** renal disease, COVID-19, viral infection, acute kidney injury

## Abstract

Kidney damage in severe acute respiratory syndrome coronavirus 2 (SARS-CoV-2) can occur even in patients with no underlying kidney disease. Signs of kidney problems can progress to a state that demands dialysis and hampering recovery. Although not without controversy, emerging evidence implicates direct infectivity of SARS-CoV-2 in the kidney. At the early stage of the pandemic, consideration was mainly on the well-recognized angiotensin-converting enzyme 2 (ACE2) receptor as being the site for viral interaction and subsequent cellular internalization. Despite the abundance of ACE2 receptors in the kidneys, researchers have expanded beyond ACE2 and identified novel viral entry pathways that could be advantageously explored as therapeutic targets. This review presents the potential involvement of toll-like receptor 4 (TLR-4), kidney injury molecule-1/T cell immunoglobulin mucin domain 1 (KIM-1/TIM-1), and cluster of differentiation 147 (CD147) in SARS-CoV-2-associated renal damage. In this context, we address the unresolved issues surrounding SARS-CoV-2 renal infectivity.

## 1. Introduction

Severe acute respiratory syndrome coronavirus 2 (SARS-CoV-2) first emerged in late 2019 and quickly spread around the word in the form of the coronavirus disease 2019 (COVID-19) pandemic, resulting in a substantial increase in morbidity and mortality [1,2]. SARS-CoV-2 has proven to be highly contagious, with more than three-quarters of a billion positive confirmed cases to date, according to the World Health Organization. Clinical data indicate that all age groups are susceptible to infection by SAR-CoV-2, yet exhibit different classes of symptoms, with those over 60 years and those with comorbidities, such as lung disease, cardiovascular diseases, diabetes, and hypertension, being more prone to develop serious fatal events [3,4]. Although acute respiratory distress syndrome and pneumonia are the main clinical manifestations of SARS-CoV-2, recently published evidence reported damage to multiple organs, including the kidneys [5,6].

An especially alarming complication of COVID-19 is abnormalities in kidney function. It has been reported that the incidence of acute kidney injury (AKI) in the setting of mild SARS-CoV-2 cases accounts for 5%, while it increases dramatically in critically ill patients [7]. For instance, a clinical perspective study on 701 patients with moderate and severe COVID-19 symptoms showed that 43.9% developed proteinuria and 13% exhibited either increased serum creatinine levels, blood urea nitrogen, or both, at hospitalization [8]. Notably, AKI has been shown to be the most prominent extra-pulmonary complication in ICU patients (29%), whereas cardiac dysfunction and liver injury account for only 23% [9]. Similarly, it has been demonstrated that the prevalence of AKI in critically ill COVID-19 patients is 20–50% higher than in those with mild and moderate symptoms [10]. Additionally, a study performed on 1248 hospitalized COVID-19 patients observed that 43% developed AKI, thereby proving a strong predictor of 30-day mortality [11].

SARS-CoV-2-related AKI can involve histopathologic characteristics, including acute tubular necrosis and podocytopathy [12,13]. The underlying mechanisms involve hemodynamic instability, disruption of the renin–angiotensin aldosterone system, and direct invasion of kidney cells by the SARS-CoV-2 [7] and the cytokine storm, which leads to the recruitment of inflammatory cells and activates apoptosis [14]. Based on published evidence, the presence of angiotensin-converting enzyme 2 (ACE2) within the cell membrane is implicated in SARS-CoV-2 entry into host cells [15], while ACE2 is highly expressed in kidneys [16], mainly in proximal tubule cells [17] and kidney podocytes [18,19]. Although ACE2 has been described as the main gateway for SARS-CoV-2, recent published evidence has demonstrated that the toll-like receptor 4 (TLR-4), kidney injury molecule-1/T cell immunoglobulin mucin domain1(KIM-1/TIM-1), and cluster of differentiation 147 (CD147) can also all serve as alternative receptors to SARS-CoV-2, thereby facilitating the entry of the virus, and aggravating kidney injury [20–23]. This review discusses what is currently known about SARS-CoV-2-induced kidney damage and any additional potential virus entry mechanisms in the observed complication.

## 2. SARS-CoV-2-Induced Kidney Abnormalities

Strong evidence has been reported that SARS-CoV2 induces kidney morphological alterations and enhances kidney dysfunction. In that regard, a study was performed on autopsies of COVID-19 postmortem patients and observed that the SARS-CoV-2 antigen accumulated in the renal epithelial tubules [5]. Similarly, a study was performed by Su *et al*. [24] on 26 kidney autopsies using electronic microscopy and found virus particles characteristic of SARS-CoV-2 mainly in the podocytes and proximal tubular epithelium, which were associated with a detachment of podocytes from the glomerular membrane. Notably, another autopsy study performed on six COVID-19 patients observed SARS-CoV-2 in the glomerulus [25]. The presence of SARS-CoV-2 particles in urine samples was also detected [26]. Collectively, these findings suggest that SARS-CoV-2 can directly induce kidney damage.

Microscopic examination on ICU patients and postmortem biopsies showed glomerular, acute tubular, and tubulointerstitial injuries along with congestion in the peritubular capillaries and glomeruli [27,28]. Furthermore, collapsing glomerulopathy has been described in patients with non-severe respiratory symptoms or those who have glomerular proteinuria. Although the molecular pathways involved in collapsing glomerulopathy post-COVID-19 are yet to be fully elucidated, this observed manifestation could be potentially mediated by podocyte injury [29,30]. Other studies showed that SARS-CoV-2 could exacerbate kidney impairment indirectly through complex immune mechanisms. For instance, Diao *et al*. [5] observed that the virus antigen accumulated in the cytoplasm of tubular cells alongside the presence of C5-b9—a complement membrane attack complex involved in the progression of tubulointerstitial damage—in the apical brush border of the epithelial cells, and a strong deposition of CD68^+^ macrophages in tubulointerstitial space, all of which suggest that the immune response could play a major role in the pathogenesis of kidney damage by COVID-19.

The most frequent clinical manifestation of kidney abnormalities in the setting of COVID-19 is AKI. Multiple studies have demonstrated increased serum creatinine levels in COVID-19 patients at admission, which is considered a negative prognostic marker of AKI [8,31–33]. It has also been observed that the majority of critically ill patients develop proteinuria and hematuria [34]. Another study on 193 COVID-19 patients observed that 59% developed proteinuria, 44% exhibited hematuria, and around 10% experienced increased serum creatinine and blood urea nitrogen levels [35]. Interestingly, no improvement in kidney function was seen in 89% of COVID-19 patients who developed AKI in a 3-week follow-up study [31]. Another clinical study reported that 46% of COVID-19 patients who exhibited AKI at discharge did not recover to baseline serum creatinine levels [33]. Therefore, further investigations are needed to evaluate the possibility that AKI in the context of COVID-19 may lead to chronic kidney diseases. Importantly, clinical data reveal a tight link between AKI and increased mortality rate post-COVID-19. In that regard, it has been shown that the mortality rate increased to 35% in patients with AKI compared to 6% for those without AKI [32].

## 3. TLR-4 Activation and Kidney Damage

Macrophages and dendritic cells express pattern recognition receptors (PPRs) either in their cytosol or on the surface in order to eliminate invading pathogens. The PPRs recognize pathogen-associated molecular patterns (PAMPs) and initiate an immune response [36]. PPRs are divided into four major families, one of which includes Toll-like receptors (TLRs) [37]. TLRs are further classified under the interleukin-1 receptor superfamily, while the first identified member was TLR-4 [38,39]. The interaction between TLR-4 and PAMPs mediates the production of proinflammatory chemokines and cytokines, which augment the inflammatory response. In addition to the proinflammatory characteristic of TLR-4, its activation can also increase type 1 interferon secretion, which is implicated in alerting other cells to the viral infection, thereby inhibiting viral replication [40]. TLR-4 has also been documented to stimulate adaptive immunity. Thus, all these mechanisms together can effectively eliminate the pathogen [41–44]. Of note, investigations have documented whereby TLR-4 has both canonical and alternative signaling pathways. The canonical pathway is mediated by myeloid differentiation primary response 88 (MyD88), which results in the production of multiple pro-inflammatory markers, whereas the alternative pathway mediates the secretion of type-1 interferon and anti-inflammatory cytokines [45,46].

TLR4 has been shown to be highly expressed by renal proximal and distal tubular epithelial cells [47–49]. These observations were further confirmed by pre-clinical experiments and clinical data. For instance, using a murine model of ischemia-reperfusion injury, epithelial cells of the kidney tubules were shown to exhibit a high expression level of TLR4 [50]. Additionally, ischemia-induced renal inflammation strongly correlates with the upregulation of TLR4 in different parts of the kidneys, such as the distal tubules, loop of Henle, and collecting ducts [51]. Furthermore, a clinical study performed on patients who underwent surgery on the urinary tract demonstrated high expression levels of TLR4 in their kidney epithelial tubular cells [52].

Multiple investigations have indicated that TLR4 is implicated in the pathogenesis of kidney disease. For example, it has been observed that the activation of TLR4 in an animal model of ischemia-reperfusion injury was associated with stimulation of the inflammatory response, subsequently, aggravating AKI [53]. TLR4 has also been shown to be expressed by the renal infiltrating leukocytes and its presence was correlated with an increase in the production of the proinflammatory cytokine IL-6, which requires an interaction between TLR4 and the high mobility group box (HMGB) protein—a nuclear protein released upon cellular damage [49]. Additionally, a study performed using C3H/HeOuJ mice showed that lipopolysaccharides (LPS) stimulated the activation of TLR4, thereby triggering the release of tumor necrosis factor-*α* (TNF-*α*), which, consequently, induced renal failure [54]. Another animal study revealed that sepsis-induced AKI was mediated by the upregulation of TLR4, resulting in enhanced neutrophil infiltration and proinflammatory cytokine production in epithelial tubular cells [55]. In cisplatin-induced kidney injury, activation of TLR was associated with enhanced leukocyte migration and tubular damage, as well as exacerbated kidney dysfunction [56,57]. In addition to the expression of TLR4 in epithelial tubular cells, another study observed that TLR4 was located in the endothelial cells and its activation was linked to the stimulation of endothelial expression of proinflammatory cytokines [58].

Another interesting mechanism implicated in TLR-induced kidney injury is the disruption of ion transport across the tubules. In that regard, TLR4 was documented to be highly expressed in the basal and lateral sides of the cells of the thick ascending limb, while its expression correlated with an alteration in bicarbonate absorption, thereby suggesting a role for TLR4 in the induction of tubular dysfunction [59]. Similarly, the TLR4/MyD88 pathway was reported to be involved in suppressing the absorption of bicarbonate and inhibiting Na^+^/H^+^ exchanger activity [60]. Furthermore, the activation of TLR4 in mice with LPS-induced kidney injuries led to a reduced urine flow rate [61]. Collectively, these findings suggest that the TLR4 canonical pathway is activated in kidney diseases.

## 4. Potential Role of TLR-4 in COVID-19

**Fig. 1.**
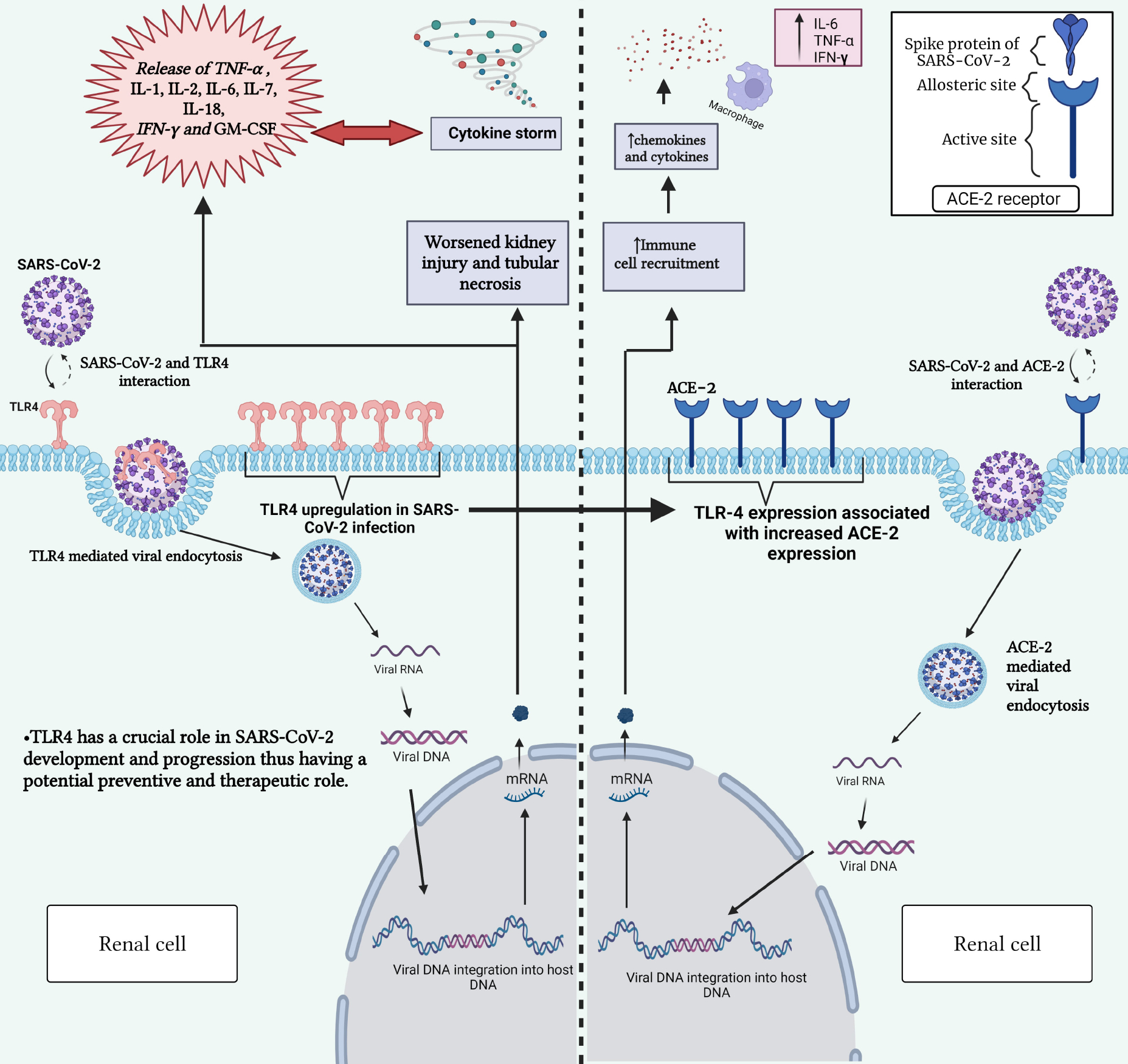
Interaction between SARS-CoV-2 and TLR-4 receptor (left) and between SARS-CoV-2 and ACE2 receptor (right). This figure highlights the role of TLR-4 in triggering immune responses upon viral recognition and the significance of ACE2 and TLR-4 receptors in mediating viral entry into host cells. Gaining insight into these interactions is vital for comprehending the mechanisms behind COVID-19 development and for devising targeted therapeutic strategies. SARS-CoV-2, severe acute respiratory syndrome coronavirus 2; ACE2, angiotensin-converting enzyme 2; TLR-4, toll-like receptor 4; COVID-19, coronavirus disease 2019; DNA, deoxyribonucleic acid; RNA, ribonucleic acid; mRNA, messenger RNA; IL-1, interleukin-1; IL-2, interleukin-2; IL-6, interleukin-6; IL-7, interleukin-7; IL-18, interleukin-18; TNF-*α*, tumor necrosis factor alpha; IFN-*γ*, interferon gamma; GM-CSF, granulocyte-macrophage colony-stimulating factor.

Recent evidence has suggested that the binding of TLR4 to the SARS-CoV-2 spike protein could serve as an alternative gateway into human cells, ultimately, aggravating the hyperinflammatory response characterized by increased TNF-*α*, interleukin (IL)-1, IL-2, IL-6, IL-7, IL-18, interferon-gamma, granulocyte-macrophages colony-stimulating factor, and monocyte chemoattractant protein-levels, known as a cytokine storm, a hallmark feature of COVID-19 (Fig. 1) [62]. In that regard, it has been documented that TLR4 has a stronger protein–protein interaction with SARS-CoV-2 compared to ACE2, with binding energy values of –120.2 and –29.2, respectively (the more negative, the stronger the binding) [63], a critical observation that requires further investigation. Another clinical study observed that S100A9, an endogenous ligand of TLR4, was highly upregulated in COVID-19 severe patients compared to healthy controls, and this increase was conversely correlated with plasma album in levels [64]. Furthermore, TLR4 along with its downstream mediators, including CD14, MyD88, and TRAF6 among others, have been observed to increase in COVID-19 patients [65]. It has also been reported that the activation of TLR4 on platelets following SARS-CoV-2 infection may explain the observed thrombotic events in COVID-19 patients, such as myocardial infarction, which potentially leads to kidney damage through the cardio–renal interrelationship, otherwise known as cardio–renal syndrome [20,66]. Leemans *et al*. [67] and Su *et al*. [68] documented an increase in TLR4 mRNA expression levels in the tubules and endothelial cells of kidneys following COVID-19, and their upregulation was correlated with enhanced tubular necrosis and an exacerbated kidney injury. A clinical study performed by Conti *et al*. [69] on COVID-19 patients showed that TLR4 was upregulated in peripheral mononuclear cells [64]. Additionally, the activation of TLRs post-COVID-19 could induce the release of the proinflammatory cytokine IL-1*β*, suggesting a crucial role for TLR4 in mediating the severity of COVID-19 [69,70]. An *in vitro* study has also reported the direct involvement of TLR4 in SARS-CoV-2-induced tubular injury via the regulation of NF-*κ*B and signal transducer and activator of transcription 3 (STAT3) pathways [71]. Collectively, TLR4 seems to play a crucial role in the development and progression of SARS-CoV-2 and has the potential to be a promising therapeutic target post-COVID-19.

## 5. KIM-1/TIM-1 and Kidney Damage

Kidney injury molecule 1 (KIM-1), previously named T-cell Ig and mucin domain 1 (TIM-1), is a type 1 membrane receptor that possesses an immunoglobulin variable Ig-like (Ig V) domain, mucin domain, a transmembrane domain, and cytosolic domain. The KIM-1 gene can undergo alternative splicing, to produce transcript variants: HAVCR1 (Hepatitis A virus cellular receptor 1) and TIM1 (T-cell immunoglobulin mucin receptor 1). HAVCR1, the earliest homolog of KIM-1, facilitates the entry of HAV, via an interaction with the IgV and mucin domains [72]. Interestingly, other studies have revealed that KIM-1 binds to many other viral families. For instance, KIM-1 binds to Ebola virus glycoproteins and phosphatidylserines, to mediate cellular entry and increase viral infectivity [73,74]. KIM-1 is also known as T-cell immunoglobulin mucin domain 1 (TIM-1), given that it co-stimulates T-cell activation, regulates Th2 reaction, and mediates cytokine formation in immune system-related diseases [75]. While barely expressed in normal kidneys, KIM-1 production becomes robust in proximal tubules in acute and chronic forms of kidney injury in rodents and humans [76]. A substantial increase in KIM-1 under conditions involving de-differentiation of epithelial cells has been frequently reported in kidney pathologies of various etiologies, including ischemia, hypoxia, and toxicity [77,78]. The alteration in KIM-1 levels happens at early stages and correlates with the kidney damage severity. Furthermore, KIM-1 undergoes membrane-proximal cleavage by metalloproteinases and shedding into the urine, which is preferentially considered a sensitive and specific biomarker of tubular injury [79,80].

Aside from its potential clinical utility, KIM-1 is thought to promote cell homeostasis and tissue remodeling in the early stage of kidney injury. It was identified by Ichimura *et al*. [81] as the first non-myeloid phosphatidylserine receptor, characterized by a phagocytic function. Through binding to phosphatidylserine (PS) epitopes on the apoptotic cell membrane, KIM-1 triggers the internalization of dead cells from the kidney tubular lumen [81]. Transient KIM-1 overexpression was also shown to repair tubular epithelial cells by promoting their migration and proliferation, plausibly influenced by the activation of the extracellular signal-regulated kinase–mitogen-activated protein kinase (ERK–MAPK) signaling pathway [82]. Moreover, the ability of KIM-1 to inhibit the binding of guanosine-5^′^-triphosphate (GTP) by G*α*_12_ via direct interaction has been demonstrated in renal ischemia reperfusion injuries (IRIs), thereby resulting in an inhibition of the activation of G*α*_12_ and any associated pathogenesis [83]. In parallel, KIM-1-deficient mice experienced more severe IRIs at the structural and functional levels in accordance with excessive G*α*_12_ activation.

Nevertheless, upon chronic expression, KIM-1 seems to reflect and contribute to sustained renal damage. This is supported by the finding that colocalization of KIM-1 and areas of *α*-SMA and macrophages were seen in the damaged proximal tubules of patients with renal diseases [84]. In experimental models of cisplatin-induced AKI, unilateral urethral obstruction, and aristolochic acid-induced renal fibrosis, high levels of KIM-1 were reported alongside the persistent elevation in renal fibrosis [85]. Using a macrophage-like cell line, Tian *et al*. [85] revealed that the MAPK pathways were involved in the TIM-1-mediated migration of macrophages and the production of M1 biomarkers, such as INF-*γ* and iNOS. Evidence showing the KIM-1 contribution to kidney pathology was revealed by the utilization of anti-TIM-1 antibodies and mutant genes. For instance, upongeneticinductionofchronicKIM-1expression in the absence of any injury in mice, extensive interstitial fibrosis, monocyte chemotactic protein-1 (MCP-1) upregulation, and MCP-1-dependent macrophages chemotaxis were prominent along with the emergence of a chronic kidney disease (CKD) phenotype by 4 weeks of age [86]. However, these alterations were absent in mice expressing mutant KIM-1 [86]. In cisplatin-induced AKI, the anti-TIM-1 antibody RMT1–10 showed protective effects by ameliorating renal histological damage and dysfunction, reducing apoptosis, and inhibiting leukocyte recruitment and inflammatory injury [87]. Concomitantly, protective measures were detected systemically where anti-TIM-1 was used to diminish CD4 and CD-68 T-cell early infiltration, apoptosis, and cytokine production [87]. Similarly, renal tubular-specific knockout of KIM-1 was found to be protective against both ischemic and cisplatin-induced AKI. The Yin Yang 1 transcription factor was identified as a negative regulator of KIM-1 expression, while KIM-1 can interact with death receptor 5 (DR5), to activate its multimerization and downstream cascade. These findings form the basis that introduced the YY1–KIM1–DR5 axis as a potential target to prevent apoptosis and injury [88]. The upregulation of KIM-1 in kidney damage is perceived to likely bridge the progression of AKI to CKD.

## 6. KIM-1/TIM-1 and COVID-19

Previously, studies have proven the presence of SARS-CoV-2 in the kidneys, particularly in epithelial cells, thereby suggesting a potential direct infectivity [23,24,89]. The IgV-domain in KIM-1 provides a means for viral invasion through its interaction with the PS constituent of the viral envelope, as demonstrated with hepatitis A and C, human immunodeficiency virus (HIV), Ebola, Dengue, and other viruses [90]. SARS-CoV-2 is known to recognize and target ACE2 receptors in the invasion process via its receptor-binding domain (RBD). KIM1 and ACE2 are both expressed in the kidney. Thus, upon renal injury, KIM-1 levels drastically increase with subtle changes in ACE2 levels [91]. Conversely, renal levels of ACE2 have been reported to decrease during SARS-CoV-2 infection [92]. Supported by the following observations, the sole responsibility of ACE2 in renal SARS-CoV-2 infection was questioned and redirected towards the prospect of KIM-1 participation. Theoretically, KIM-1 seems capable of creating a vicious cycle manifested by injury-induced KIM-1 expression.

Laboratory parameters examined in 102 SARS-CoV-2 patients illustrated high levels of KIM-1 at admission, which increased alongside the disease severity [93]. In severe courses, Chen *et al*. [89] featured high vulnerability of renal distal tubules to SARS-CoV-2 infection. The levels of KIM-1 in the distal tubules of these patients were remarkably upregulated and accompanied by inflammatory accumulations of MCP-1, interleukin 6 (IL-6), etc. [89]. Mori *et al*. [23] also showed that KIM-1 was expressed in the proximal tubules of the biopsies from three SARS-CoV-2 patients with AKI and in 14 of 30 post-mortem kidney samples from patients with documented SARS-CoV-2 infection [15]. Interestingly, enhanced KIM-1 expression was also shown to be associated with a reduced expression of ACE2. Functional approaches that demonstrate KIM-1 as causative for SARS-CoV-2 revealed a role for renal KIM-1 in endocytosis of the viral particles, which was prevented by the use of anti-KIM-1 antibodies and TW-37, a newly discovered inhibitor of KIM-1-mediated endocytosis [23]. The uptake of spike-conjugated virosomes by human epithelial KIM-1 expressing “tubuloids” was noticed and further increased by overexpressing KIM-1 [23].

**Fig. 2.**
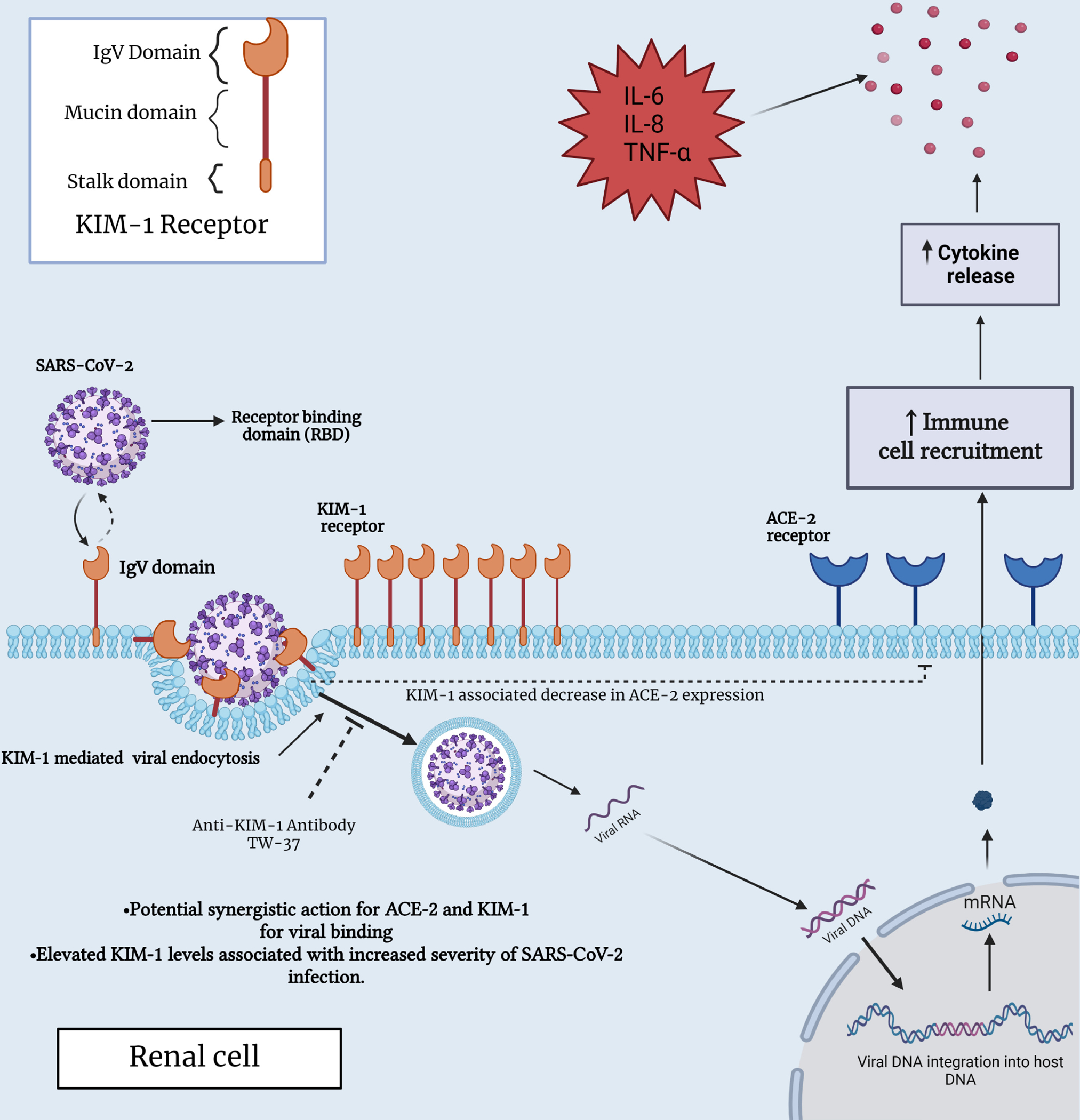
Interaction between SARS-CoV-2 and KIM-1 receptor in a renal cell. This figure provides in sights into the molecular interplay between KIM-1 and SARS-CoV-2, shedding light on their potential significance in both immune and renal cell biology, specifically, within the context of COVID-19 infection. ACE2, angiotensin-converting enzyme 2; IL-6, interleukin-6; IL-8, interleukin-8; TNF-*α*, tumor necrosis factor alpha; IgV, immunoglobulin domain; KIM-1, kidney injury molecule-1; DNA, deoxyribonucleic acid; RNA, ribonucleic acid; mRNA, messenger RNA.

The interaction between KIM1 and SARS-CoV-2 was investigated by Yang *et al*. [91], who characterized the binding pocket, affinity, and attachment with validations using rationally designed KIM1-derived antagonist peptide polypeptide 2 (AP2). The interaction was further confirmed by co-immunoprecipitation and fluorescence resonance energy transfer (FRET)-based assays using cell lysates from human kidney tubular epithelial cell lines HK-2 and HEK293T. These findings demonstrated the crucial involvement of the KIM-1 IgV domain in viral recognition and binding. A lower binding free energy was observed for KIM-1 compared to ACE2, and by referring to the divergence in binding sites on the RBD virus between KIM-1 and ACE2, a synergistic action for these receptors was speculated [91] (Fig. 2).

These findings provide promising insights for using KIM-1 as a therapeutic target, given the constitutive expression and site unspecific features of ACE2. Further studies that elucidate the relationship between these receptors in SARS-CoV-2 and that consider the burden of KIM-1 in patients with and without AKI are of the utmost importance.

## 7. CD147 and Kidney Damage

CD147, also known as Basigin (BSG) or extracellular matrix metalloproteinase inducer (EMMPRIN), is a multifunctional transmembrane glycoprotein, which is classified in the immunoglobulin superfamily [94]. It is widely distributed in a variety of organs, including the brain, liver, spleen, and kidneys, and is expressed in many cell types, including hematopoietic, epithelial, and endothelial cells [94]. CD147 comprises an extracellular region with two immunoglobulin domains: a transmembrane domain and a cytoplasmic domain [95,96]. Three organ-dependent N-linked glycosylation sites were identified in the extracellular domain [97], contributing to the multifunctional properties of CD147. These functions reflect multiple interacting partners of CD147, including caveolin-1, cyclophilins, monocarboxylate transporters (MCTs), CD147 itself, integrins, and E-selectins [98–101], while the signaling pathways involved via CD147 include the MAPK cascade, PI3-kinase, ERK1/2, and NF-*κ*B, which are involved in cell apoptosis, proliferation, and are the product of cytokines and MMPs 1 [102–104].

Numerous biological roles have been associated with CD147. At the immunological level, CD147 is suggested to act as a potential negative regulator of T-cell activation [105], as well as a receptor for viral entry in host cells in the context of HIV [106]. Additionally, at the inflammatory level, CD147 has been shown to be engaged in inflammatory diseases, such as cardiac infarction, atherosclerosis, AKI, and renal fibrosis [101,107–109].

CD147 is known to be highly expressed in kidneys through a growing body of evidence, which has revealed the distribution of CD147 in normal kidneys, notably in the distal and proximal tubular epithelial cells (TECs) [110], although not in the glomerular components [111]. CD147 is thought to be involved in the pathophysiology of kidneys, especially in AKI, which is amplified by inflammatory cell infiltration through the release of chemotactic cytokines and reactive oxygen species accompanied by ATP depletion due to leukocyte–endothelial cell interaction [112].

Consistent with the above, the interaction between CD147 and its ligand cyclophilin A (CyPA) is assumed to be critical in the regulation of leukocyte recruitment during inflammation. Mounting evidence has demonstrated that extracellular cyclophilin exerts proinflammatory effects via CD147, whereas anti-CD147 antibodies are anti-inflammatory. Several *in vivo* studies, including with AKI, supported this concept. Indeed, Dear *et al*. [113] revealed that intraperitoneal injections of anti-CD147 antibodies inhibited CD147, the receptor of CyPA, which in turn attenuated renal dysfunction. Furthermore, administration of anti-CD147 antibodies reduced the production of proinflammatory cytokines such as TNF-*α*, thereby suggesting that CD147 inhibition could prevent AKI [113]. Moreover, E-selectin promotes leukocyte secretion during inflammation. Kato *et al*. [101] demonstrated the underlying effect of CD147 and E-selectin interaction as an inducer of renal inflammation in ischemia/reperfusion; thus, demonstrating that neutrophil migration, a key contributor in AKI, resulting from E-selectin binding to CD147, was suppressed in CD147-deficient mice, resulting in a decreased tubular injury. Additionally, energy consumption was reported to be correlated with AKI [114]. It has been demonstrated that MCTs, another ligand of CD147, play a pivotal role in transporting monocarboxylates, such as lactate and pyruvate, across plasma membranes by acting as a fuel for ATP production [115]. The presence of a chaperone molecule, including CD147, is primordial for their anchorage and activity at the plasma membrane [98], a finding confirmed by their co-localization [115]. As far as renal injuries are concerned, recent evidence found that ATP depletion in patients with AKI was strongly correlated with a deficiency in CD147. Moreover, hypoxia-induced ATP depletion was prominent in TECs of CD147-deficient mouse models [110]. These results favor the hypothesis that the interactivity of CD147 and MCTs might participate in the metabolism of lactate, which is a function of CD147 in AKI.

## 8. CD147 and COVID-19

**Fig. 3.**
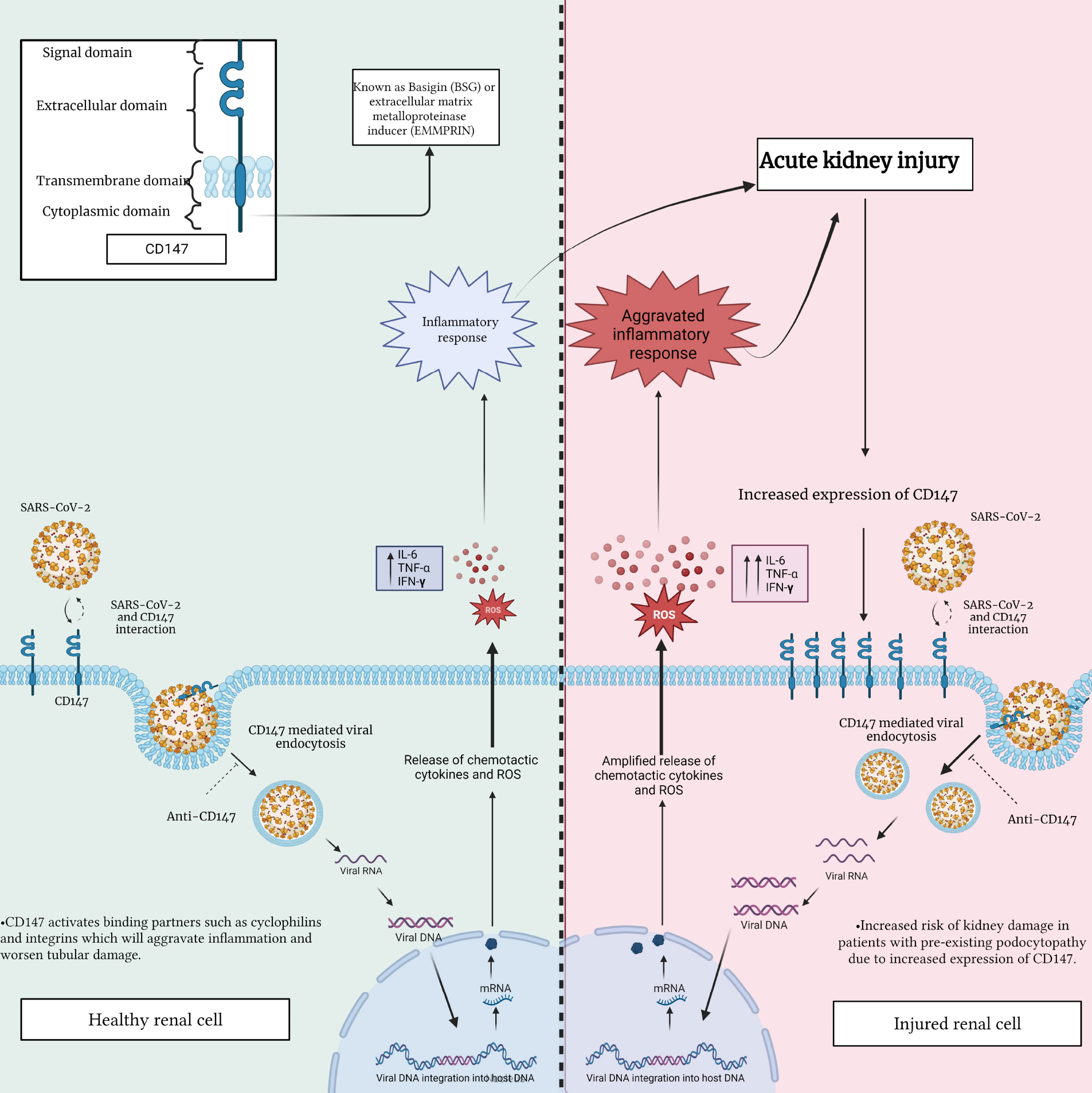
Interaction between SARS-CoV-2 and CD147 in a healthy renal cell compared to an injured renal cell. This figure highlights the potential differences in CD147 expression and distribution, emphasizing the importance of comprehending the impact of this interaction on renal cell function. This understanding is crucial in assessing the potential consequences of renal injury and disease during SARS-CoV-2 infection. IL-6, interleukin-6; TNF-*α*, tumor necrosis factor alpha; ROS, reactive oxygen species; IFN-*γ*, interferon-gamma; CD147, cluster of differentiation 147; DNA, deoxyribonucleic acid; RNA, ribonucleic acid; mRNA, messenger RNA.

CD147 has been indicated to be a potential novel entry route for SARS-CoV-2. Wang *et al*. [116] observed via immunoelectron microscope that CD147 localized with the spike protein in SARS-CoV-2-infected Vero E6 cells; thus, mediating viral entry into the kidney epithelium. As previously mentioned, CD147 is highly expressed on the basolateral side of tubular epithelial cells in normal kidneys [110]. In the context of COVID-19, SARS-CoV-2 penetrates renal proximal tubular epithelial cells through the luminal surface and the basolateral side. The aforementioned findings in the previous section provide insight into the possibility that SARS-CoV-2, associated with CD147, targets renal tubules by entering from the basolateral side, subsequently activating CD147 binding partners, such as cyclophilins and integrins [24], which aggravates inflammation and worsens renal tubular damage [117] (Fig. 3).

Yoshioka *et al*. [118] reported that CD147 is rarely expressed in healthy podocytes, meaning podocytes could be a target for SARS-CoV-2 entry, while SARS-CoV-2-induced podocyte injury is mediated by the increased expression of CD147 [119]. In this regard, viral inclusion within the vacuoles of the podocyte was revealed by electron microscopy in a patient with AKI after SARS-CoV-2 infection [120]. Additionally, Gupta *et al*. [121] confirmed COVID-19-mediated podocytopathy, by reporting on the transition from minimal change disease to collapsing glomerulopathy in a patient with an initial clinical presentation of nephrotic syndrome. As mentioned earlier, in the podocytes of the glomerulus, CD147 is only expressed on the injured podocyte, while the extensive expression of CD147 might facilitate the entry of SARS-CoV-2 and increase injury in patients with a high risk of podocytopathy [122]. Interestingly, Kalejaiye *et al*. [123] indicated that an antibody blockade of CD147 receptors mitigated SARS-CoV-2 viral uptake in human-induced pluripotent stem cell-derived kidney podocytes. Overall, CD147 appears to be highly correlated with the onset of COVID-19, thereby providing evidence for using CD147 as a new potential therapeutic strategy in the prevention and treatment of SARS-CoV-2 infections in human tissues and organs.

## 9. Controversies

AKI is a common complication of COVID-19, while CKD is a high-risk factor for COVID-19 and its related mortality [124]. Transcriptome analysis has identified common pathways and molecular biomarkers for COVID-19, AKI, and CKD [125]. These findings were echoed in a larger study on renal biopsies from patients with COVID-19, which showed that a major pathological renal feature from COVID-19 resonated as an acute tubular injury [126]. However, whether the SARS-CoV-2 virus does in fact infect kidney cells in order to replicate remains controversial. Uptake of spike-conjugated virosomes by KIM-1 has been described in a preprint; thus, has not undergone peer review. In addition, early electron microscopic images that purportedly show the coronavirus in the kidneys of COVID-19 patients have been disputed [127,128]. Alternatively, Radovic *etal*. [129] provided histopathological and immunofluorescence evidence of SARS-CoV-2 infection and viral replication along with tissue injury of renal parenchymal and tubular epithelial cells in patients with severe COVID-19. Nonetheless, while kidney organoids and cultured cells are easily susceptible to SARS-CoV-2 infections, the kidneys of COVID-19 patients appear to be somewhat resistant. This observation raises the possibility that they may be injured through some indirect mechanism that is influenced by the host response, genetic susceptibility, physiological disturbances, or therapies [130]. Alternatively, some genetic factors may enhance the susceptibility of the kidney to SARS-CoV-2 infection.

Moreover, it remains a possibility that the receptors discussed in our review are upregulated after infection, and thus, modulate the disease process. Such may be the case in particular with regard to KIM-1, which seems to suppress or enhance inflammation in a ligand-dependent manner. KIM-1 reduces inflammation by downregulating NF-*κ*B when it endocytoses or phagocytoses phosphatidylserine or apoptotic cell bodies [81,131], whereas KIM-1 enhances inflammation and fibrosis when it endocytoses fatty acid-bound albumin, which is abundant in formative urine [132]. Thus, it may be speculated that the ligand-dependency of KIM-1 is associated with the pathogenesis of COVID-19 when it endocytoses SARS-CoV-2.

## 10. Conclusions and Future Direction

Emerging research is rapidly expanding our knowledge of the pathophysiology of SARS-CoV-2 and its interaction with human cellular receptors. The new evidence suggests that in addition to the known ACE2 receptor, TLR-4, KIM-1/TIM-1, and CD147 play crucial roles in the pathogenesis of COVID-19 and specifically in the mediation of viral entry into kidney cells and the onset of kidney cell damage. Future studies investigating the role of these receptors should be conducted to identify their potential involvement in the development of long-COVID, in addition to their possible implications in the progression to CKD. In the future, the development of therapeutic drugs with specific antagonist properties to these receptors might diminish the inflammatory response of the pathogen and pave the way for the development of more effective therapies for COVID-19. Extensive clinical research has yielded compelling evidence regarding the efficacy of specific medications in combatting COVID-19. These medications have shown favorable outcomes in effectively managing the disease and minimizing its associated complications, as supported by robust clinical studies (Table 1).

Long-COVID, also referred to as post-acute sequelae of SARS-CoV-2 infection (PASC), pertains to a variety of persistent and often chronic symptoms that some patients endure after recovering from the acute phase of COVID-19 infection [133]. Globally, it is estimated that around 10% of COVID patients are afflicted with long-COVID (https://www.who.int/europe/news-room/fact-sheets/item/post-covid-19-condition) [133]. Some of the most common symptoms of long-COVID include chest pain, heart palpitations, joint pain, headaches, and cognitive difficulties [134]. Furthermore, new research has shown that individuals with long-COVID may have a higher likelihood of developing CKD [135,136]. Patients with pre-existing CKD are also at greater risk of severe COVID-19 infections, which can damage multiple organs and trigger an exaggerated inflammatory response [137].

**Fig. 4.**
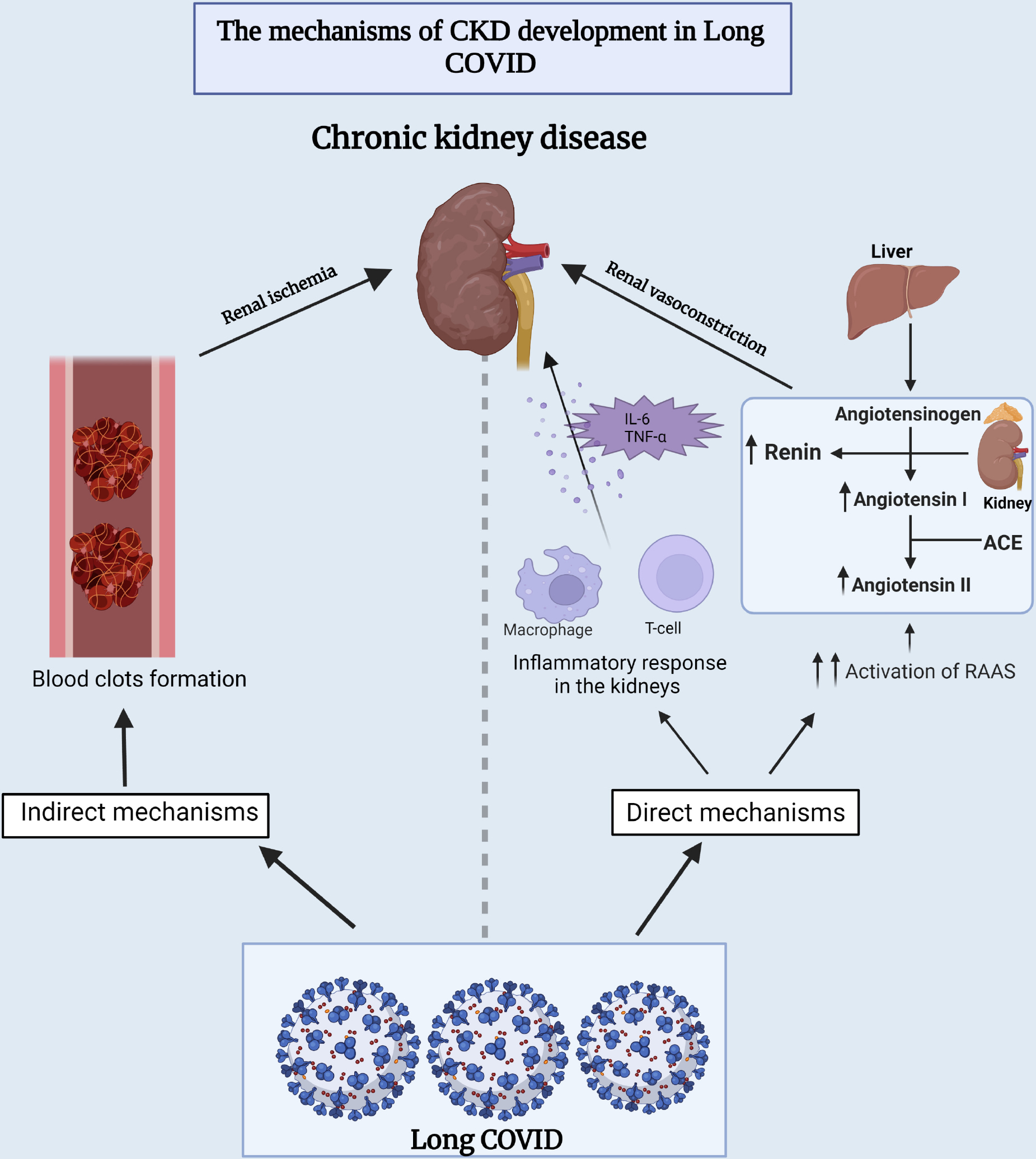
Underlying mechanisms of chronic kidney disease development during long-COVID. This figure provides insights into both the direct and indirect mechanisms that contribute to the development of chronic kidney disease in individuals experiencing long-term COVID-19 symptoms. ACE, angiotensin-converting enzyme; RAAS, renin-angiotensin-aldosterone system; IL-6, interleukin-6; TNF-*α*, tumor necrosis factor alpha.

Several theories have been proposed to understand the fundamental mechanism between long-COVID and CKD. One is that long-term COVID-19 may cause direct renal damage by infiltrating inflammatory cells or through the overactivation of the renin-angiotensin-aldosterone (RAAS) system, which is thought to enhance kidney damage over time [138]. The other hypothesized mechanism is through the formation of blood clots, which in turn leads to ischemia in the kidneys and subsequent tissue damage [138] (Fig. 4).

Another important complication associated with long-COVID is postural orthostatic tachycardia (POTS) [139]. POTS is an intricate disorder that is characterized by orthostatic symptoms, such as lightheadedness, palpitations, fainting, brain fog, and tachycardia without orthostatic hypotension, all of which have a considerable negative impact on quality of life [140,141]. It is estimated that around 2% to 14% of all COVID patients develop POTS [139]. However, the precise ways in which long-COVID pertains to POTS remain uncertain. It is speculated that damage to the autonomic nervous system through direct toxicity and the invasion of the central nervous system by SARS-CoV-2 could be major underlying factors in the development of POTS [142]. However, evidence of unchanged plasma renin activity and low aldosterone in the face of reduced plasma volume, and a red blood cell volume deficit in patients with POTS, indicate that the kidneys also play a key role [143].

## Figures and Tables

**Table 1.** Clinical trials investigating the potential role of TLR-4 antagonists and anti-CD147 in COVID-19 patients.

Drug name	Mechanism of action	Study national clinical trial number	Number of patients	Allocation	Current phase of the study

ERITORAN	TLR4 antagonist	NCT02735707	10,000	Randomized	Phase 3 COVID-19 trial
EB05	TLR4 antagonist	NCT04401475	396	Randomized	Phase 2 COVID-19 trial
RESVERATROL	TLR4 antagonist	NCT04622865	45	Randomized	Phase 2 COVID-19 trial with zinc therapy
CURCUMIN	TLR4 antagonist	NCT04382040	50	Randomized	Phase 2 COVID-19 trial for a medical spray
					ArtemiC comprising artemisinin, curcumin. frankincense, and vitamin C
		NCT04468139	60	N/A	Phase 4 COVID-19 trial for quadruple therapy (zinc, quercetin, bromelain, and vitamin C)
QUERCETIN	TLR4 antagonist	NCT04377789	447	Randomized	N/A
ISOQUERCETIN	TLR4 antagonist	NCT04622865	200	Randomized	Phase 2 COVID-19 trial
	Anti-CD147	NCT05679492	1320	Randomized	Phase 3 COVID-19 trial
	Anti-CD147	NCT04586153	456	Randomized	Phase 2 COVID-19 trial
MEPLAZUMAB	Anti-CD147	NCT05679479	350	Randomized	Phase 3 COVID-19 trial
	Anti-CD147	NCT05113784	150	Randomized	Phase 2 COVID-19 trial
	Anti-CD147	NCT04275245	17	N/A	Completed
